# A cell-based model of pathological tau uptake and propagation

**DOI:** 10.1186/1750-1326-8-S1-P17

**Published:** 2013-09-13

**Authors:** Ben Falcon, Rachel Angers, Michel Goedert

**Affiliations:** 1 MRC Laboratory of Molecular Biology, Cambridge, UK

## Background

Increasing cognitive impairment and neuronal loss correlate with the spread of tau inclusions through the brain. When present in large quantities, these inclusions contribute to Alzheimer’s disease and other neurodegenerative diseases termed tauopathies. [1]. More recently, experiments both *in vitro* and *in vivo* have observed that pathological tau can transfer between cells and transmit a misfolded state to cytosolic soluble tau [2]. We have developed a cell-based model to dissect the mechanisms behind the propagation of tau pathology and assess potential therapeutic strategies.

## Materials and methods

Filamentous tau from Tg mice expressing P301S tau or filamentous recombinant P301S tau, with or without DyLight^®^ 488 label, were added to HEK 293T cells expressing soluble P301S tau. The uptake of exogenous tau and induced fibrillization of endogenous tau was monitored by flow cytometry, immunofluorescence and SDS-PAGE-WB. Specific inhibitors, markers and kinetic experiments were used to examine the mechanisms of tau internalization and interaction within cells. Truncated tau constructs were expressed in cells to assess the structural requirements for induced fibrillization.

## Results

Hyperphosphorylated filamentous tau from P301S tau mice and non-phosphorylated filamentous recombinant P301S tau both efficiently entered cells and induced the formation of hyperphosphorylated filamentous tau composed of endogenous protein, in a time- and concentration- dependent manner. By labelling filamentous recombinant P301S tau with DyLight^®^ 488, we were able to monitor cellular uptake iand interaction with endogenous tau. This revealed that whilst the majority of cells internalized exogenous tau filaments, only a subset of tau expressing cells retained these and formed inclusions composed of endogenous protein. Studying the kinetics of exogenous tau uptake, along with inhibitors of cell binding and endocytosis, suggested that this process depends on the exogenous tau filaments binding to cell surface proteoglycans and being taken up by a mechanism most consistent with macropinocytosis. We were not able to induce tau fibrillization in cells expressing a N-terminal tau fragment, or tau with fibril forming motif deletions. However, induced tau fibrillization was observed when expressing a C-terminal tau fragment, or solely the repeat region.

## Conclusions

We developed and optimized a cell-based model of pathological tau propagation, whereby the addition of minute quantities of exogenous filamentous tau induces the formation of intracellular hyperphosphorylated filamentous tau by direct interaction with endogenous soluble tau. Using this model we have been able to study mechanisms underlying this process. We have shown that cell surface binding and endocytosis are vital for the uptake of filamentous tau and the subsequent induction of endogenous tau misfolding and propagation to other cells. The expression of truncated tau constructs revealed that the repeat region of tau and the ability to form filaments are necessary for induced fibrillization. This model could be a potential tool to assess mechanism-based therapies.

